# Polymorphisms of the Dopamine D4 Receptor Gene in Stabled Horses are Related to Differences in Behavioral Response to Frustration

**DOI:** 10.3390/ani3030663

**Published:** 2013-07-26

**Authors:** Shigeru Ninomiya, Akiko Anjiki, Yudai Nishide, Minori Mori, Yoshitaka Deguchi, Toshiyuki Satoh

**Affiliations:** 1 Faculty of Applied Biological Sciences, Gifu University, 1-1 Yanagido Gifu 501-1193, Japan; 2 Department of Veterinary and Medicine Science, Tokyo University of Agriculture and Technology, 3-5-8 Saiwai, Fuchu, Tokyo 183-8509, Japan; E-Mails: anko_o3@yahoo.co.jp (A.A.); tsatoh@cc.tuat.ac.jp (T.S.); 3 Locust Research Laboratory, National Institute of Agro-biological Sciences at Ohwashi, Tsukuba, Ibaraki 305-8634, Japan; E-Mail: nishiyu0@yahoo.co.jp; 4 Faculty of Agriculture, Iwate University, Morioka, Iwate 020-8550, Japan; E-Mails: marino20961@yahoo.co.jp (M.M.); deguchi@iwate-u.ac.jp (Y.D.)

**Keywords:** animal welfare, behaviour, frustration, DRD4 gene, SNPs, stabled horse

## Abstract

**Simple Summary:**

In this study, the association between behavioral responses to frustration and DRD4 polymorphisms was investigated in horses. Twenty one horses were observed for 4–4.5 h during feeding for several days. Horses were then genotyped for the DRD4 gene. Behavioral responses to frustration were recorded more frequently in horses without the A allele than in horses with the A allele. These results indicate that polymorphisms of DRD4 should be considered when assessing the welfare of stabled horses, by means of behavioral indicators of frustration.

**Abstract:**

In stabled horses, behavioral responses to frustration are often observed, especially around feeding time. These behavioral responses are a useful indicator of their welfare. In this study, we investigated the association between this behavioral indicator and DRD4 gene polymorphisms in stabled horses. Twenty one horses housed in two stables were used. The horses were observed for approximately 4 h around feeding over three or more days using focal-sampling and instantaneous-sampling. Horses were genotyped for the A–G substitution in the DRD4 gene. The effects of the A–G substitution (with or without the A allele in the DRD4 gene), the stables, and their interaction on the frequency of behavioral responses to frustration were analyzed using general linear models. The total time budget of behavioral responses to frustration was higher in horses without the A allele than in those with the A allele (P = 0.007). These results indicate that the A–G substitution of the DRD4 gene is related to frustration-related behavioral responses in stabled horses. Appropriate consideration should be made for the DRD4 gene polymorphism when the welfare of stabled horses is assessed, based on this behavioral indicator.

## 1. Introduction

When debating how farm animal welfare should be regarded in society, we require an objective and scientific assessment of welfare. Farm animals live in an environment that is provided and managed by humans. Animals in the farm environment will experience stress if a stockperson does not control and manage some aspects of the environment well (see, for example, ‘Five Freedoms’ [1]). In this context, animals try to cope with the stressor using behavioral, physiological, or neurological functions [2]. Behavior is an effective coping mechanism for animals. In this coping phase, behavioral needs [3] arise in animals, and the animals are motivated to perform appropriate behaviors for coping with a stressor. However, when farm animal behavior is restricted by intensive housing, such as cages, stalls, or high-density housing, there is a high probability of the animal being dissatisfied with the behavioral need. Situation such as these generate frustration in animals. Some behavioral responses, such as displacement activity and abnormal behavior, are then typically expressed in these situations [4]. These behaviors can be used as indicators of animal welfare [5].

In stabled horses, abnormal behaviors (e.g., stereotypic behavior [6,7]), displacement behavior (e.g., pawing; [7,8]), and appetitive behaviors (e.g., bedding investigation behavior [7,9,10]) are reported as negative behavioral indicators. However, standing-sleep behavior is reported as a positive behavioral indicator [10]. These behaviors have been used as scientific indicators of horse welfare [10,11,12,13,14,15]. Prior studies have found that these behavioral responses to frustration differ among breeds in horses. Epidemiological studies have revealed that the prevalence of stereotypic behavior differs across breeds [16,17,18]. It was also reported that the time budget of bedding investigation behavior was higher in thoroughbreds than in the other breeds [7].

Behavioral genetic research has identified polymorphisms in the dopamine D4 receptor (DRD4) gene associated with horse temperament [19]. In the study, horse temperament was assessed by a caretaker administering a questionnaire survey. Differences in temperament scores of ‘curiosity and vigilance’ were found in relation to an A–G substitution in the DRD4 gene. These authors proposed that dopamine is likely involved in the expression of behavior in active coping strategies [20]. In fact, the expression of equine stereotypy is linked with the dopamine receptor [21,22,23].

For this study, we hypothesized that the A–G substitution in the DRD4 gene is involved in behavioral response in some motivational states, including frustration. In addition, horses frequently show behavioral responses to frustration around feeding time [7,24]. Therefore, we tested whether the presence of the A–G substitution in the DRD4 gene was related to behavioral responses to frustration around the feeding time of stabled horses.

## 2. Experimental Section

### 2.1. Subjects

Subjects were 21 horses (18 geldings and three mares) ranging in age from 4 to 23 years (mean age 14 years; S.D. 5.1). Horses that intensely engaged in cribbing were not used, because stereotyped cribbing would be a self-rewarding behavior [22] and established stereotypies tend to be expressed independent of external factors [25]. The horses were thoroughbred (*n* = 19), thoroughbred mixed breed (*n* = 1), and Oldenburg (*n* = 1). Twelve horses were housed at the Tokyo University of Agriculture and Technology (group T). Nine horses were at Iwate University (group I). All horses were kept individually in box stalls. Drinking water was available *ad libitum*. The boxes were cleaned daily. In group T, horses received a pelleted concentrate and hay at 05:00, 12:00, and 16:30. They were ridden and trained in the morning. In group I, horses received a pelleted concentrate and hay at 08:30, 12:00, and 17:00 and received hay at 21:00. They were ridden and trained from 05:00 to 08:00.

This study was conducted according to the Fundamental Guidelines for Animal Experimentation and Related Activities at each university.

### 2.2. Behavioral Observation

Behavioral categories included eating (eating feed or bedding), resting (lying down or standing with one hind leg lifted), standing (standing without other behavior), moving, defecating, urinating, drinking, looking outside (looking outside the stall with ears turned forward), bedding investigation (smelling or removing bedding with its nose), pawing, licking or biting (licking or biting a stall structure), head-shaking (shaking the head up and down), weaving (swinging the body and head from side to side), crib-biting (bending the neck with grasping, or not, a stall structure with the incisors, sometimes accompanied by a distinctive sound), and other behavior.

In group T, the behavior of each horse was observed during 12:00–16:30 for three days each in May, August, and November 2009, and was instantaneously recorded at 3 min intervals [26]. For management reasons, the data on a given day were excluded from the analysis if a horse was mostly out of its box during the observation period. If a horse was out of its box briefly, the behavior time budget was calculated based on the periods when the horse was in its box. In group I, the behavior of each horse was observed during 10:00–14:00 for three days from May–July 2010, and was instantaneously recorded at 1 min intervals.

### 2.3. Investigation of Polymorphisms in the DRD4 Gene

In group A, hair was drawn from the mane of each horse. Genomic DNA was isolated from each hair sample using a kit (ISOHAIR; Nippon Gene Co. Ltd., Toyama, Japan). In group B, a blood sample was drawn from the jugular vein of each horse. Genomic DNA was isolated from each blood sample using a DNeasy Tissue Kit (Qiagen Inc., Tokyo, Japan). Genetic analyses were conducted according to the previous study of the DRD4 A–G substitution [19]. The genetic analysis revealed that 13 horses were A present type and 8 horses were A absent type.

### 2.4. Data Analysis

The total time budget (%) allocated to behavioral responses to frustration—which were weaving, crib-biting, licking or biting, pawing, head shaking, and bedding investigation—was calculated for individual animals on each observation day. Furthermore, the time budgets of eating, looking outside, and resting were also calculated. Data of all observation days were averaged for individual animals. Data were assessed to ascertain whether the assumptions of parametric testing had been met. Data of looking outside were angular-transformed for normal distribution. General linear models were applied using MINITAB version 14 (Minitab Inc., State College, PA). These behavioral data were used as dependent variables. In the model, polymorphism (with A allele or without A allele), group (T or I), and their interaction were fixed factors.

## 3. Results and Discussion

The total time budget of behavioral responses to frustration was higher in horses without the A allele than in horses with the A allele (*F*_1,17_ = 9.21, *P* = 0.007). There were no statistically significant differences between horses with and without the A allele in any other behavioral measure (Table 1). All results suggest that A–G substitution in the DRD4 gene does not influence the overall activity around feeding time in stabled horses.

**Table 1:** Mean time budgets (% ± SE) of each behavioral category in horses with and without the A allele in DRD4 gene.

Behavioral category	with the A allele			without the A allele		
behavioral responses to frustration	9.5	±	1.9	20.5	±	3.0
eating	41.4	±	3.3	29.5	±	4.0
looking outside	11.8	±	1.9	9.4	±	1.9
resting	28.2	±	3.5	33.4	±	3.1

There were also differences between groups in the time budget of eating (Group I = 46.5% (SE = 3.5), Group T = 29.6% (SE = 2.7), *F*_1,17_ = 4.58, *P* = 0.047). There were no other significant differences between groups and there were no significant interactions. The finding of a significant difference in eating between groups was expected to result from differences in the observation periods or how horses were managed, e.g., the amount of feed in each group. 

Previous studies have found that factors such as the feeding regime, quality of feed, access to posture, type of bedding, and box design had effects on the expression of behavioral response to frustration [9,11,13,16,17,18,27,28]. It may be that horses in this study tried to cope with frustration related with these factors. In such a situation, they would be motivated to perform some normal behaviors (e.g., bedding investigation behavior as appetitive behavior of eating [7,9,10,11]); displacement (e.g., pawing), redirected (e.g., licking or biting a stall structure), or abnormal behavior (e.g., stereotypic behavior) would also be expressed. 

An earlier study of DRD4 polymorphisms in horses showed that the A–G substitution influenced two temperament traits, assessed via a questionnaire [19]. Horses without the A allele were seen by caretakers as being more prone to approach novel objects and being less vigilant about surroundings than those with the A allele. A study of mice also indicated that the DRD4 gene was associated with responsiveness in behavioral tests [29]. These previous studies and the present study indicate that the A–G substitution of the DRD4 gene is associated with behavioral responses in some motivational states in stabled horses.

The results of this study also suggest that when the welfare of stabled horses is assessed via behavioral responses to frustration, it is necessary to take the DRD4 gene polymorphism into account. Not doing so risks confusing genetic differences in behavioral responses to frustration with the actual welfare state of stabled horses. However, we cannot discount the alternative possibility that horses without the A allele might be more frustrated in the stable than horses with the A allele. Furthermore, it is also necessary to investigate the effect of the gene on the other aspects of their welfare because animal welfare comprises both physical and mental health. 

## 4. Conclusions

A difference in behaviors related to frustration was related to the A–G substitution of the DRD4 gene in stabled horses. DRD4 polymorphisms should be considered when assessing the welfare of stabled horses. 
